# Distinguishing neurocognitive deficits in adult patients with NP-C from early onset Alzheimer’s dementia

**DOI:** 10.1186/s13023-018-0833-3

**Published:** 2018-06-05

**Authors:** Andreas Johnen, Matthias Pawlowski, Thomas Duning

**Affiliations:** Department of Neurology, University Hospital of Münster, Albert-Schweitzer-Campus 1, Building A1, 48149 Münster, Germany

**Keywords:** Niemann-Pick disease type C, Alzheimer’s disease, Dementia, Cognitive function

## Abstract

**Background:**

Niemann-Pick disease type C (NP-C) is a rare, progressive neurodegenerative disease caused by mutations in the *NPC1* or the *NPC2* gene. Neurocognitive deficits are common in NP-C, particularly in patients with the adolescent/adult-onset form. As a disease-specific therapy is available, it is important to distinguish clinically between the cognitive profiles in NP-C and primary dementia (e.g., early Alzheimer’s disease; eAD).

**Methods:**

In a prospective observational study, we directly compared the neurocognitive profiles of patients with confirmed NP-C (*n* = 7) and eAD (*n* = 15). All patients underwent neurocognitive assessment using dementia screening tests (mini-mental status examination [MMSE] and frontal assessment battery [FAB]) and an extensive battery of tests assessing verbal memory, visuoconstructive abilities, visual memory, executive functions and verbal fluency.

**Results:**

Overall cognitive impairment (MMSE) was significantly greater in eAD vs. NP-C (*p* = 0.010). The frequency of patients classified as cognitively ‘impaired’ was also significantly greater in eAD vs. NP-C (*p* = 0.025). Patients with NP-C showed relatively preserved verbal memory, but frequent impairment in visual memory, visuoconstruction, executive functions and in particular, verbal fluency. In the eAD group, a wider profile of more frequent and more severe neurocognitive deficits was seen, primarily featuring severe verbal and visual memory deficits along with major executive impairment. Delayed verbal memory recall was a particularly strong distinguishing factor between the two groups.

**Conclusion:**

A combination of detailed yet easy-to-apply neurocognitive tests assessing verbal memory, executive functions and verbal fluency may help distinguish NP-C cases from those with primary dementia due to eAD.

## Background

Niemann-Pick disease type C (NP-C) is a rare, progressive neurodegenerative disease caused by mutations in the *NPC1* or the *NPC2* gene, which lead to impaired cholesterol metabolism [1, 2]. Neurocognitive and neuropsychiatric deficits are commonly reported in patients NP-C, particularly among patients with the adolescent/adult-onset form [1, 3–5]. Early-onset cognitive decline (EOCD) is characterised by presentation of cognitive impairment before the age of 65 years, and clinical diagnoses of EOCD are increasingly being reported. Most cases are related to early Alzheimer’s disease (eAD). However, compared to dementia in patients aged > 65 years, there is a much wider range of differential diagnoses, including underlying inherited neurodegenerative aetiologies such as NP-C.

NP-C shares a number of clinical and neuropathological features in common with AD and other dementias, including eAD, frontotemporal dementia (FTD), and Lewy body dementia [6–10]. Patients with EOCD and eAD have therefore been suggested as a potential clinical niche for the identification of new, as yet undetected cases of NP-C [11]. In particular, patients with dementia-plus syndromes featuring concomitant psychiatric symptoms, movement disorders such as degenerative ataxia and/or vertical supranuclear saccade palsy (VSSP) are also considered to have an increased likelihood of having NP-C [11–13].

Although the burden of clinical symptoms of adult NP-C patients impacts on activities of daily living, cerebral magnetic resonance imaging (MRI) findings are usually normal or show non-specific minor cerebral atrophy [2, 5]. Thus, cognitive deterioration among patients with adolescent/adult-onset NP-C can easily be mistaken for primary psychiatric disorders or other neurodegenerative dementias [12–14]. It is therefore important to be able to distinguish at the initial clinical level between the cognitive profile of patients with NP-C and those with common dementia subtypes such as eAD. However, there are few published studies on valid neurocognitive tests that could be used to distinguish adult NP-C patients from those with other neurodegenerative dementia aetiologies [15].

A wide range of neurocognitive symptoms are encountered among NP-C patients, but published data on the specific neurocognitive profile of NP-C are based on studies adopting a range of methodologies, resulting in inconsistent or incomplete findings [12, 16–19]. Early signs of cognitive impairment have been reported to comprise reduced executive function, processing speed, and verbal memory due to frontal-subcortical neural dysfunction [3, 17, 19]. Continued disease progression leads to further general cognitive decline, progressive deterioration in abilities to perform daily tasks, highly diminished memory, and behavioural impairment [5, 17, 20, 21].

Based on findings from a pilot study in 10 patients with NP-C, Klarner et al. [17] reported impairments in fine motor skills, language, attention, working memory, and visuospatial functions. A number of deficits similar to those seen in eAD were observed, including verbal episodic memory impairment. Appropriate neurocognitive tests incorporating disease staging were recommended to detect NP-C: the Trail Making tests (TMT) A & B and verbal fluency tests were judged as most useful in patients with mild disease, and The Mini-Mental Status Examination (MMSE), Corsi Block-tapping, Find Similarities, and Clock Drawing Tests were considered more appropriate in patients with more advanced disease. Other, separate studies have reported frontal impairments and decreases in executive functions and attention [5, 16]. A systematic literature review of case reports based on 23 separate patients has also reported executive dysfunction as the most frequent cognitive deficit in adult NP-C [15], possibly due to neuropathology in cerebellar regions and deep grey matter nuclei (e.g., the thalamus and striatum). Overall, the current literature points towards attentional and executive dysfunctions as the hallmark of neurocognitive impairment in patients with NP-C. However, it remains unclear whether the previously recommended tests assessing these cognitive domains could help distinguish NP-C patients from other dementia subtypes such as eAD.

The recognition of NP-C as early as possible in the disease course is important as a current treatment (miglustat) is available in Europe and many other countries that may help stabilise the progression of neurological and neurocognitive symptoms. Intrathecal hydroxypropyl-beta-cyclodextrin is a promising future alternative treatment that is currently undergoing clinical trials. Encouraging data with this agent were reported by Ory et al. in 2017, and further studies are ongoing [14, 16, 22–25].

The advent of biochemical laboratory markers and the increasing availability of next-generation gene sequencing methods are major advances for the identification of new cases of NP-C. However, these methods are only applied in patients or patient groups considered at high risk of NP-C. Clinical methods of defining the level of risk or suspicion of NP-C are necessary to direct such further diagnostic investigations. The characterization and delineation of the neurocognitive profile of NP-C may help clinicians to achieve earlier diagnoses, particularly when combined with approaches that help distinguish potential cases from another primary degenerative disease such as eAD.

In this study, we used a detailed neurocognitive test battery to assess whether there is a recognizable, specific profile of deficits in adult patients with genetically proven NP-C. We were particularly interested in whether neurocognitive tests from attentional and executive domains identified by previous studies as useful in the detection of NP-C (e.g., TMT-A and B, verbal fluency tests) were able to differentiate NP-C from eAD. Importantly and in contrast to previous studies [17], the neurocognitive test battery used here was geared towards the typical age span of patients with adult NP-C and eAD instead of senile dementia, in which ceiling effects must be expected. Thus, we conducted the first direct comparison of the observed NP-C neurocognitive profile with a reference group of adults with confirmed eAD, the most frequent aetiology of EOCD.

## Methods

### Study design

This was a prospective observational cohort study including consecutive patients encountered at the Memory Disorder Unit at the Department of Neurology, University Hospital Münster, Germany between 2012 and 2016. All participants had confirmed pathogenic *NPC1* genotypes.

Included patients underwent neurological and psychiatric examination by a physician trained in the assessment of patients suffering from dementia. Lumbar puncture, electroencephalography, event-related potentials (P300), 3.0 Tesla brain MRI, and comprehensive neurocognitive testing were also performed. Within the eAD group, all patients were classified as having probable eAD according to McKhann criteria [26], with at least intermediate pathophysiological evidence of AD based on cerebrospinal fluid biomarker profile and MRI brain atrophy pattern. All patients underwent follow-up visits for ≥12 months. Functional disability was evaluated in NP-C patients using a well-established and validated disease-specific disability scale for NP-C [27], which assesses six key domains (ambulation, manipulation, language, swallowing, ocular movements, and epilepsy) on a composite scale with scores ranging from 0 (best) to 24 (worst).

Subjects with a history of neurological disorders other than eAD or NP-C such as other dementia subtypes, other neurodegenerative disorders (e.g., Huntington’s disease, multiple system atrophy, motor neuron disease), stroke, hydrocephalus, epilepsy, brain tumour, traumatic brain injury, major psychiatric illness not related to dementia (e.g., drug or alcohol abuse), and other systemic diseases known to interfere with cognitive function were excluded.

### Assessments

Demographic data and clinical parameters were collected for all patients. An extensive battery of neurocognitive tests was applied, which assessed all major cognitive domains. The test battery included: the German equivalent of the Rey Auditory Verbal Learning Test (RAVLT) [28] for verbal span, verbal learning efficiency, verbal short-term retrieval, verbal long-term retrieval, and verbal recognition; the Rey Complex Figure Test and Recognition Trial (RCFT) [29] for visuoconstructive abilities and visual short-term retrieval; TMT-A and -B for processing speed, and set-shifting speed, respectively [30]; the Regensburger verbal fluency test (RWT) [31] for lexical and semantic word fluency; and the forwards and backwards digit span tests from the Wechsler Memory Scale (WMS) [32] for attention and working memory capacity. Among these neurocognitive parameters, lower raw scores indicate better performance on the TMT-A, TMT-B, RCFT time to copy, the RAVLT5–6, and RAVLT5–7 scores. Higher raw scores signify better performance for all other parameters.

Overall cognitive function was screened using the MMSE [33], where higher scores represent less impairment. Frontal-executive impairment was screened using the Frontal Assessment Battery (FAB), where higher scores also indicate less impairment [34].

### Data analysis

Between-group differences were evaluated using the Welch t-test for normally distributed data, and the non-parametric Mann-Whitney test for non-normally distributed data. Raw scores from all neurocognitive tests were transformed into normative percentile ranks stratified by age, gender and education, where cognitive ‘impairment’ was defined when patients scored below the 10th percentile rank (PR) of the respective normative sample detailed in the professional manuals for each test. The proportions of patients categorized as ‘impaired’ based on their neurocognitive profiles were compared between the NP-C and eAD groups using the Chi-square test. For all between-group comparisons, statistical significance was concluded at the *p* < 0.05 level.

Cohen’s *d* effect-sizes for between-group differences, taking into account the within-group variance and homogeneity of scores, were derived for each test parameter. Based on this analysis, receiver operating characteristics (ROC) curves were used to calculate the sensitivity and specificity of the parameter with the largest effect-size for differential diagnosis between NP-C and eAD, and in test parameters previously suggested to be useful in detecting mild NP-C [17].

## Results

### Patients

A total of nine patients with NP-C were selected for inclusion in the study, all of whom had confirmed diagnoses based on molecular genetic testing. Two NP-C patients who were initially selected for the study were excluded from the data analysis because they were not testable due to the severity of their motor dysfunction. Individual patient demographics, disease characteristics, biochemical variables and disability scores of the seven included NP-C patients are presented in Table 1. All seven patients had been symptomatic (symptom duration 6–14 years) and were on ongoing miglustat treatment for a range of 3–14 months. Ataxia was the most common symptom at initial presentation (three patients), followed by cataplexy and dysarthria (in two patients each). None of the patients in this cohort showed more than mild dementia, and no patients presented with epileptic seizures or psychotic symptoms. Disability scores indicated relatively mild disease severity [27].

**Table 1:** Demographic and disease characteristics of patients with NP-C

Demographics			Clinical data					Biochemical variables			
Patient No.	Age (yrs)	Gender	Age at onset (yrs)	Symptom duration (yrs)	Duration of miglustat therapy (mo)	Primary clinical symptoms^a^	NP-C severity score^b^	Plasma ChT (nmol/h/mL)	Plasma C-triol (ng/mL)	CSF Aβ (pg/mL)	CSF tau (pg/mL)
1	35	F	28	7	10	Ataxia^a^, dysarthria, mild VSGP	6	56	112	ND	ND
2	31	M	17	14	26	Dysarthria^a^, psychomotor agitation, dysphagia, ataxia, VSGP	14	321	267	1337	807
3	45	F	34	11	18	Cataplexy^a^, ataxia, dysarthria, dystonia, mild VSGP	11	321	108	789	151
4	27	F	17	6	37	Cataplexy^a^, mild ataxia and dysarthria	5	97	91	ND	ND
5	54	F	42	12	28	Ataxia^a^, dysarthria, VSGP, mild dysphagia	9	367	302	ND	ND
6	33	F	25	8	3	Dysarthria^a^, mild VSGP, mild ataxia	7	77	164	1282	313
7	23	F	14	9	22	Ataxia^a^, dystonia, mild dysarthria, VSGP, cataplexy, mild dysphagia	10	128	188	1267	354

^a^Symptoms present at initial presentation; ^b^NP-C severity measured using the NP-C disability scale of Pineda et al [27], where escores ranged from 0 (best) to 24 (worst). Aβ, amyloid-beta protein; *CSF* cerebrospinal fluid, *ChT* chitotriosidase, *C-triol* cholestane-3β,5α,6β-triol, *ND* not determined. Analyte cut-off values: ChT < 100 nmol/h/mL; C-triol < 50 ng/mL; Aβ < 500 pg/mL; tau > 500 pg/mL

Table 2 shows a comparison of NP-C and eAD patient group characteristics. The mean ± SD age of the adult/adolescent onset NP-C patients in this cohort (34.6 ± 12.2 years) was significantly lower than that in the reference group of eAD patients (55.1 ± 3.4 years; *p* = 0.004). Patients in both groups had a similar level of education.

**Table 2:** Comparison of patient demographics and clinical characteristics in NP-C and eAD patients

Parameter	NP-C patients (*n* = 7)	eAD patients (*n* = 15)	Group difference
			*p*-value
Age in years, mean ± SD	34.6 ± 12.2	55.1 ± 3.4	*p* < 0.01
Male: female, n (%):	2 (22): 7 (78)	6 (40): 9 (60)	–
Years in education, mean ± SD	11.6 ± 1.5	11.3 ± 1.5	*p* = 0.68
Amyloid-ß level (pg/ml)^a^, mean ± SD	1168 ± 255	550 ± 148	*p* < 0.01
Total tau level (pg/ml)^a^, mean ± SD	406 ± 281	860 ± 337	*p* = 0.03

^a^Amyloid and tau based on *N* = 4 NP-C patients

Disease biomarker levels in the eAD group confirmed that all eAD patients had notable/substantial AD pathology. Amyloid-ß and total tau levels were measured in four NP-C patients. Mean total tau concentration was significantly higher in the eAD group compared with NP-C patients (*p* = 0.03), and amyloid-ß levels were significantly lower among eAD patients (*p* < 0.01) (Table 2).

### Neurocognitive test results

Dementia screening indicated significantly greater overall cognitive impairment among eAD patients compared with NP-C patients based on MMSE scores (*p* = 0.010). While no statistical difference in frontal-executive impairment between NP-C and eAD patients was observed based on FAB screening (*p* = 0.245).

**Fig. 1:**
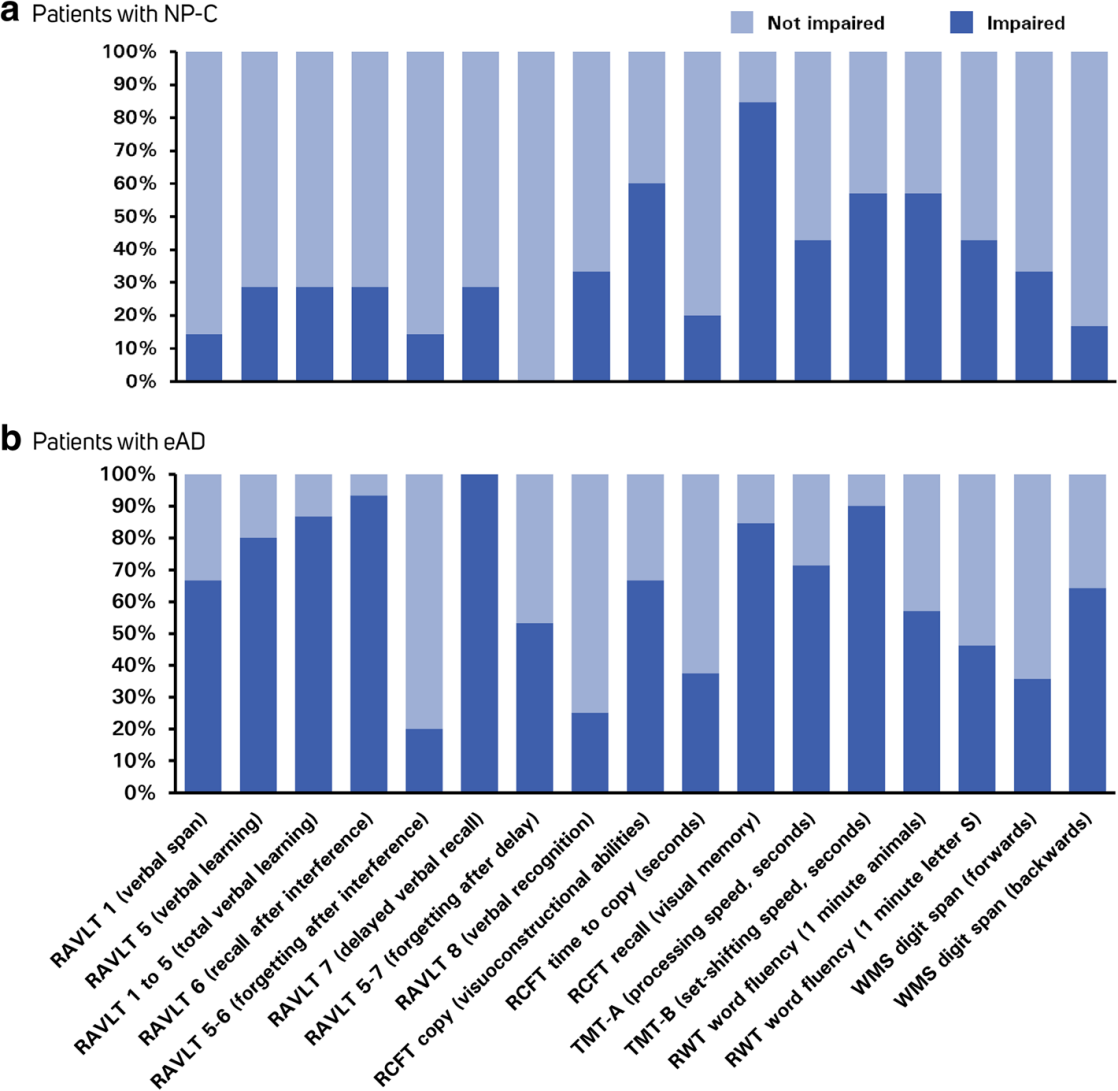
Neurocognitive profiles of patients with **a** NP-C and **b** eAD. Patients with eAD generally showed a wide profile of cognitive impairment with marked memory deficits, while patients with NP-C showed relatively preserved verbal memory (RAVLT), but frequent impairments in visuoconstruction, visual memory (RCFT recall [visual memory] and RCFT copy scores), and set-shifting and verbal fluency. RAVLT, Rey Auditory Verbal Learning Test; RCFT, Rey Complex Figure Test and Recognition Trial; RWT, Regensburger verbal fluency test; TMT, Trail-Making Tests A and B; WMS Wechsler Memory Scale

The frequencies of cognitive impairment in relation to data from age-matched normative samples for both patient groups are summarised in Fig. 1. Overall, patients with NP-C showed a cognitive profile of relatively preserved verbal episodic memory (RAVLT), but visuoconstruction and visual memory (RCFT recall [visual memory] and RCFT copy scores) were frequently impaired. Deficits in attention and executive functions (i.e., processing speed [TMT-A], set-shifting [TMT-B], and RWT word fluency) were also common. In contrast, patients with eAD generally showed a wider profile of cognitive impairment. Deficits in verbal learning and memory (RAVLT), visuoconstruction and visual memory (RCFT), and executive functions (TMT and verbal fluency scores) were similarly or more frequent than in the NP-C group.

Overall, Chi-square analysis showed that impaired test parameters (i.e., those with performance scores PR < 10) were significantly more frequent in the eAD group compared with the NP-C group (*p* = 0.025). The largest discrepancies in the frequency of cognitive impairments between the NP-C and eAD groups were in terms of verbal memory deficits (all RAVLT parameters), which were substantially more frequently observed in eAD patients.

Mean ± SD neurocognitive test raw scores are summarised in Table 3. In the majority of neurocognitive test parameters, significantly greater average impairment was seen in eAD compared with the NP-C group. Significant between-group differences indicated greater degrees of impairment in eAD patients in most RAVLT subscales (*p*-values from < 0.001 to 0.018), the RCFT copy subscale (*p* = 0.038) and the WMS digit span backwards test (*p* = 0.037). Notably, the TMT-B score, which has been highlighted as a particularly suggestive test for executive dysfunction in NP-C, was also significantly more impaired in the eAD group versus the NP-C group (*p* = 0.017).

**Table 3:** Dementia screening and neurocognitive test parameter scores in patients with NP-C and patients with eAD

Test	n	NP-C patients (*n* = 7)	n	eAD patients (*n* = 15)	Mean group difference	Cohen’s *d* effect-size^a^	*p*-value^b^
		Mean ± SD		Mean ± SD			
Dementia screenings							
MMSE	4	27.0 ± 2.16	13	21.9 ± 1.9	5.1	2.5	*p* = 0.010
FAB	5	14.4 ± 3.3	5	11.8 ± 3.3	2.6	0.8	*p* = 0.245
Neurocognitive test parameters							
RAVLT1 (verbal span)	7	7.0 ± 3.1	15	3.3 ± 1.6	3.7	1.5	*p* = 0.018
RAVLT5 (verbal learning)	7	12.9 ± 3.1	15	6.7 ± 2.7	5.6	1.9	*p* = 0.002
RAVLT1–5 (total learning)	7	51.0 ± 14.6	15	28.6 ± 8.7	22.4	1.9	*p* = 0.006
RAVLT6 (recall after interference)	7	10.1 ± 4.4	15	3.3 ± 2.5	6.9	1.9	*p* = 0.005
RAVLT5–6 (forgetting after interference)	7	2.1 ± 1.8	15	3.5 ± 1.9	−1.3	− 0.7	*p* = 0.141
RAVLT7 (delayed verbal recall)	7	10.7 ± 3.5	15	1.9 ± 2.1	8.8	3.0	*p* < 0.001
RAVLT5–7 (forgetting after delay)	7	1.6 ± 1.1	15	4.8 ± 2.1	−3.2	−1.9	*p* < 0.001
RAVLT8 (recognition)	7	13.7 ± 1.9	14	11.3 ± 3.9	2.4	0.5	*p* = 0.058^†^
RCFT copy (visuoconstructional ability)	5	29.8 ± 4.1	13	20.7 ± 12.9	9.1	0.9	*p* = 0.038
RCFT time to copy (seconds)	5	233.4 ± 95.9	8	306.5 ± 123.5	− 73.1	−0.7	*p* = 0.259
RCFT recall (visual memory)	5	12.3 ± 8.0	13	4.8 ± 4.8	7.5	1.1	*p* = 0.107
TMT-A (processing speed; seconds)	7	61.6 ± 40.7	14	93.1 ± 81.5	−31.5	−0.3	*p* = 0.252^†^
TMT-B (set shifting; seconds)	7	119.0 ± 54.4	10	226.7 ± 101.9	− 107.7	−1.3	*p* = 0.017
RWT word fluency (1 min animals)	7	17.3 ± 5.1	13	14.8 ± 5.4	2.4	0.5	*p* = 0.335
RWT word fluency (1 min letter S)	7	8.4 ± 3.1	14	11.4 ± 7.8	−3.0	−0.5	*p* = 0.225
WMS digit span (forwards)	6	7.0 ± 2.4	14	5.9 ± 1.4	1.1	0.6	*p* = 0.353
WMS digit span (backwards)	6	5.7 ± 1.5	14	3.8 ± 1.9	1.9	1.1	*p* = 0.037

^a^Effect sizes calculated according to methodology for Cohen’s d statistic for paired t-tests; ^b^*p*-values specified based on the Welch t-test, unless otherwise specified; ^†^*p*-values based on the Mann-Whitney test; For TMT-A, TMT-B, RCFT time to copy, RAVLT5–6, and RAVLT5–7 subscales, lower scores indicate better performance. Higher raw scores signify better performance for all other neurocognitive test parameters. For dementia screening scores, higher scores represent less impairment on the MMSE and the FAB. *FAB* Frontal Assessment Battery, *MMSE* Mini-Mental Status Evaluation, *RAVLT* Rey Auditory Verbal Learning Test, *RCFT* Rey Complex Figure Test and Recognition Trial, *RWT* Regensburger verbal fluency test, *TMT* Trail-Making Tests A and B, *WMS* Wechsler Memory Scales

No other cognitive tests showed significant between-group differences. However, NP-C patients showed a marginally (albeit non-significantly) worse average performance on the RWT phonematic word fluency (letter S) test compared with eAD patients (*p* = 0.225).

**Fig. 2:**
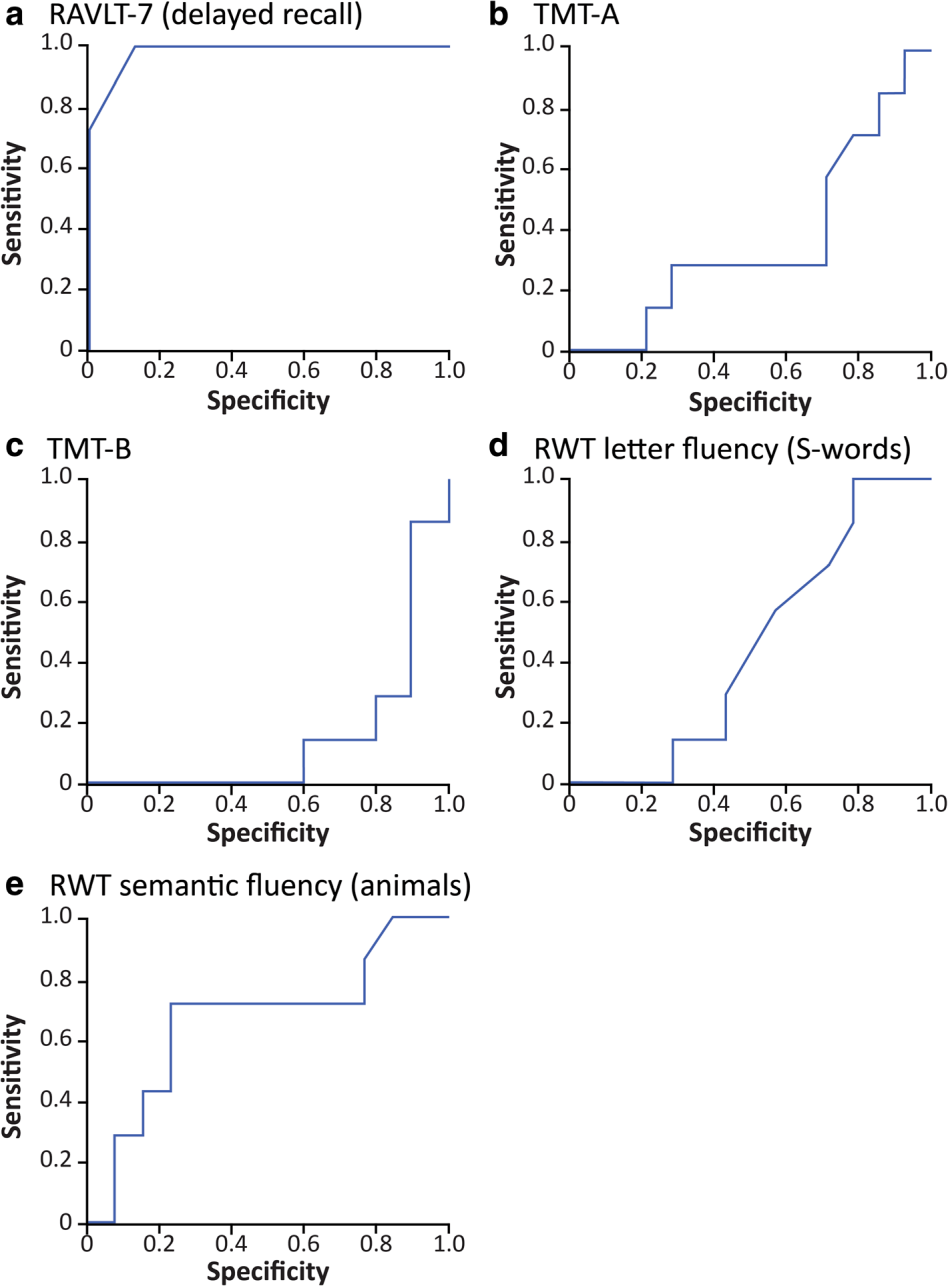
Receiver operating curves (ROC) analyses of key neurocognitive test raw scores in eAD and NP-C patients. A high AUC for the RAVLT-7 but low to moderate AUCs for the TMT-A, TMT-B, RWT letter fluency (S-words) and RWT semantic fluency (animals) were observed. Area-under-curve values: **a** 0.981 for RAVLT-7; **b** 0.362 for TMT-A; **c** 0.143 for TMT-B; **d** 0.444 for RWT letter fluency (S-words); **e** 0.665 for RWT semantic fluency (animals). ROC, Receiver operating characteristics curve analysis; RAVLT, Rey Auditory Verbal Learning Test; RWT, Regensburger Word Fluency Test; TMT, Trail Making Test

Among all of the neurocognitive tests we applied, the greatest difference between the NP-C and eAD groups in terms of deficit frequency was in RAVLT7 (delayed verbal recall) scores. This parameter also showed the greatest between-group effect size in terms of raw scores. ROC analysis showed that this parameter discriminated well between patients with NP-C and those with eAD, with an area under the curve (AUC) of 0.981 demonstrating both high sensitivity (85.7%) and high specificity (93.3%) using a cut-off of 6/15 recalled words after 30 min. Other neurocognitive tests, including those previously suggested as effective in detecting mild NP-C, showed insufficient discriminative power (all AUC values < 0.7) (Fig. 2).

Importantly, there did not appear to be any influence of neurological deficits (e.g., speech or manipulation impairment) on patients’ performance of neurocognitive tests. Subscores for these domains on the NP-C disability scale assessments showed only slight to moderate impairements: all subscores were ≤ 3 for all patients.

## Discussion

This is the first study to directly compare the neurocognitive symptom profile of patients with NP-C with that seen in a ‘reference’ group of patients with deficits due to eAD – the most common primary neurodegenerative dementia syndrome before the age of 65 years. The detailed battery of neurocognitive tests applied in this study identified specific, statistically significant differences between the NP-C and eAD groups, which may aid in the differential diagnosis of patients with early cognitive impairment and further neurological deficits (i.e., dementia-plus syndrome) suggestive of possible NP-C.

NP-C and AD share a number of pathophysiological similarities such as increased levels of brain tau protein, amyloid deposition, the presence of neurofibrillary tangles, and the influence of apolipoprotein E ε4 genotype [6–9, 35]. Other neurological commonalities include basal forebrain cholinergic system alterations and chronic neuroinflammation [36–38]. However, there are distinct differences in the localisation of neuropathology between the two conditions. Purkinje cells in the cerebellum are the most affected neurons in NP-C, with NFTs mainly found in subcortical structures, while lesions in AD are mainly seen in the neocortical and medial temporal lobes [6, 15, 39, 40]. It therefore follows that certain clinical neurocognitive differences exist between NP-C and eAD patients [16, 17].

MRI and diffusion tensor imaging (DTI) studies in NP-C patients have suggested that cerebral atrophy in key deep grey matter regions including the hippocampus, thalamus, cerebellum, and striatum, as well as major white-matter tracts, may account for global impairments in cognitive function in NP-C [41–43]. More specifically, positron emission tomography (PET) studies in adult NP-C patients have indicated that frontal-lobe hypometabolism may contribute to frontal-executive deficits [44, 45].

Overall, taking patient age, gender, and education into account, eAD patients showed wider, more generalised impairments in affected cognitive domains compared with the profile seen in NP-C patients in the current study. Deficits were also more frequent and greater in magnitude in eAD on most of the neurocognitive tests that we employed. Bergeron et al. reported that in contrast to NP-C, the general cognitive profile in AD is characterized predominantly by memory dysfunction [15]. In the current study, the key neurocognitive difference between NP-C and eAD was also observed in terms of verbal memory performance, as measured by the RAVLT. Most RAVLT subscores (excluding forgetfulness after interference) were statistically significantly more impaired in eAD patients. In particular, the RAVLT7 subscale (delayed verbal recall), an indicator of verbal memory that has been shown to correlate with hippocampal pathology [46, 47], showed the greatest effect size between the two groups. Our ROC analyses suggested that delayed verbal recall also showed good sensitivity and specificity in distinguishing between NP-C and eAD patients. Relatively preserved verbal memory has recently also been reported in a French cohort of 21 patients with mild adult NP-C [16].

With regard to alternative tests for verbal episodic memory, the Consortium to Establish a Registry for Alzheimer’s disease (CERAD) word list recall test also addresses this neurocognitive domain through delayed recall of a learned word list and is faster to apply than the RAVLT. However, the disadvantage of this test is that it is geared toward dementia patients of higher age, and normative data for younger patients are not available [17]. The Free and Cued Selective Reminding Test (FCSRT) is an alternative verbal memory test that, similar to the RAVLT, provides normative data versus younger adults, and might therefore also be suited to cognitive assessments in NP-C [16].

A number of previous studies in patients with NP-C have described impaired executive functions (e.g. set-shifting and word fluency) and attention as the primary neurocognitive deficit in NP-C [5, 15–17, 19]. Our findings are in line with previous published evidence, in that executive functions as measured by the TMT-A/B and word fluency as measured by the RWT, were the most frequently impaired domains in NP-C patients [17]. However, the frequency and magnitude of these impairments were actually lower among NP-C patients on the majority of neurocognitive tests assessing attention and executive functions versus eAD. As a result, these tests cannot be expected to function as differential cognitive markers on their own.

Interestingly, the RWT word fluency (letters) task was the only neurocognitive measure on which NP-C patients were more impaired than eAD patients at the raw score level. However, the NP-C versus eAD effect-size seen with this parameter was small and non-significant, and the frequency of impaired cases (compared with age-matched normative data) on this test was similar in both groups.

While global dementia screening tools such as the MMSE, or more specific tools such as the FAB may not be powerful enough to distinguish specific neurocognitive deficits between NP-C and eAD, the majority of NP-C patients in our study presented with impaired performance on both of these scales. In particular, FAB may prove useful for this purpose in clinical practice as it effectively assesses executive dysfunctions in a time-economic way. Overall, due to lack of specificity and the lack of normative age-matched control data in younger patients, these screening tests cannot substitute an extensive neurocognitive assessment, particularly in addressing memory and executive function.

The small sample size, particularly of the NP-C group is a weakness of this study. However, this is due to the extreme rarity of NP-C and the single-centre nature of this study. The between-group differences in age in the NP-C and eAD patients introduces a further degree of uncertainty, with patient age acting as a potential confounding factor in our statistical analyses. Age has an important influence on the range and severity of cognitive, neurological, visceral and psychiatric symptoms in NP-C [1, 2, 15]. It is notable that patients with eAD in this study were significantly older than those with NP-C. While this could be considered as a study limitation, it should also be noted that, in general, cognitive impairment in NP-C has a lower age at onset compared with eAD. While cognitive deterioration is observed in most patients with adolescent/adult-onset NP-C [1, 2, 15], EOCD is most usually recognized among patients with childhood-onset NP-C in the form of intellectual developmental disorder, poor school performance, and learning disabilities [4, 15, 48]. One difficulty in this respect is that not all neurocognitive tests are similarly applicable in different age-groups, particularly in patients with the juvenile-onset or early adolescent-onset forms: this constitutes a problem. For instance, Klarner et al. used the CERAD battery to determine the cognitive profile of patients with NP-C [17]. However, this test battery is not associated with normative data for patients aged < 50 years and frequently produces ceiling effects in younger patients. In contrast, the RCFT, RAVLT, TMT, RWT and WMS-digit span tests included in the current study all provide normative data for both children, as well as older age groups. The current test battery may therefore be more suited for use in NP-C than classical senile dementia test batteries like the CERAD or dementia screenings.

Finally, structural neuroimaging data to investigate potential links between memory scores and medial temporal lobe atrophy in eAD versus NP-C, and the possible relationship between frontal executive functions and frontal/subcortical atrophy with executive dysfunction, are a subject for potential, future studies. In addition, more specific cognitive and behavioural functions associated with frontal lobe integrity (e.g., social cognition tests, abnormal behaviour) may also function as differential markers for NP-C. However, these aspects have not yet been tested in detail in this rare patient group.

## Conclusion

In conclusion, based on our findings, we recommend that general dementia screening assessments (e.g., MMSE, FAB) should not be used in isolation to evaluate cognitive performance in patients with suspected NP-C, as these patients may present with milder cognitive deficits than patients with eAD. Such screening studies may therefore prevent the detection of important differences in the neurocognitive profiles between eAD and NP-C. Detailed, yet easy-to-apply neurocognitive tests such as the RAVLT assessing verbal episodic memory, the TMT-B assessing set-shifting and a verbal letter fluency task, with normative data available for age-groups typically associated with NP-C, appear more appropriate. Further studies in larger numbers of patients are warranted. Parallel studies of such neurocognitive scales alongside imaging parameters (e.g., based on MRI volumetry or DTI) would allow further insight through the assessment of underlying neuroanatomical correlates of cognitive dysfunction in NP-C.

## Abbreviations

AUC: Area under the curve
DTI: Diffusion tensor imaging
eAD: Early Alzheimer’s disease
EOCD: Early-onset cognitive decline
FAB: Frontal Assessment Battery
FAB: Frontal assessment battery
FCSRT: Free and Cued Selective Reminding Test
FTD: Frontotemporal dementia
MMSE: Mini-mental status examination
MRI: Magnetic resonance imaging
NP-C: Niemann-Pick disease type C
PET: Positron emission tomography
PR: Percentile rank
RAVLT: Rey Auditory Verbal Learning Test
RCFT: Rey Complex Figure Test and Recognition Trial
ROC: Receiver operating characteristics
RWT: Regensburger verbal fluency test
TMT-A/TMT-B: Trail Making tests A & B
WMS: Wechsler Memory Scale
